# Shortening of treatment duration in patients with chronic hepatitis C genotype 2 and 3 - impact of ribavirin dose - a randomized multicentre trial

**DOI:** 10.1186/1756-0500-4-220

**Published:** 2011-06-29

**Authors:** Andreas Maieron, Sigrid Metz-Gercek, Thomas-Matthias Scherzer, Hermann Laferl, Gabriele Fischer, Martin Bischof, Michael Gschwantler, Peter Ferenci

**Affiliations:** 1 Gastroenterology & Hepatology, Elisabethinen Hospital, Fadingerstrasse 1, Linz, 4020, Austria; 2 Hygiene, Microbiology and Tropical Medicine, Elisabethinen Hospital, Fadingerstrasse 1, Linz, 4020, Austria; 3 Gastroenterology & Hepatology, Internal Medicine III, Medical University Vienna, Waehringergürtel 18 - 20, Vienna, 1090, Austria; 4 Internal Medicine 4, Sozialmedizinisches Zentrum Süd - Kaiser-Franz-Josef Hospital, Kundratstraße 3, Wien 1100, Austria; 5 Department of Psychiatry and Psychotherapy, Medical University Vienna, Waehringer Guertel 18-20, Vienna 1090, Austria; 6 Internal Medicine 4, Hospital Rudolfstiftung, Juchgasse 25, Vienna, 1030, Austria; 7 Internal Medicine 4, Wilhelminenhospital, Montleartstraße 37, Vienna, 1160, Austria

**Keywords:** Hepatitis C, treatment duration, Ribavirin dose, genotype 2 and 3, randomized controlled trial

## Abstract

**Background:**

Chronic hepatitis C (CHC) Patients, infected with genotype (GT) 2 or 3 are treated with Peg-IFN and ribavirin (RBV) (800 mg/day) for 24 weeks. Treatment duration can be shortened to 12-16 weeks if a higher dose of RBV (1.000/1.200 mg/day) was used without considerable loss of responsiveness or increased risk of relapse. Previously we have shown that in patients with CHC, GT 2/3 RBV can be reduced to 400 mg/day if administered for 24 weeks without an increase in relapse rates. Therefore we investigated the efficacy of a reduced RBV dosage of 400 mg/day with shorter treatment duration (16 weeks).

**Methods:**

Treatment naïve patients with CHC, GT 2/3 were randomized to receive 180 μg peginterferonα2a/week in combination with either 800 (group C) or 400 mg/d (group D) for 16 weeks. The primary endpoint was SVR.

**Results:**

12 months after the first patient was randomized a inferior outcome of group D as compared to group C was noted, therefore the study was terminated. At study termination 89 patients were enrolled (group C: 31, D: 51). The SVR rate was statistically different in the two study groups with 51.6% in group C and 28.4% in group D (p = 0.038). Patients with low viral load had higher SVR rates (C: 67%, D: 33%) than those with high viral load (C: 33%, D: 21%).

**Conclusion:**

Both treatment duration and the dose of RBV play a major role to optimize outcome of patients with GT3. If one intends to shorten the treatment weight based RBV dose should be used, if lower RBV doses are used patients should be treated for at least 24 weeks as. A treatment regimen with a reduced RBV dosage and shortened treatment duration is associated with low SVR rates due to high relapse rates.

**Trial registration:**

NCT01258101

## Background

Chronic hepatitis C is one of the leading causes of chronic liver disease in Europe. Eight to ten million individuals suffer from chronic hepatitis C and about 20% will develop liver cirrhosis and hepatocellular carcinoma within the next 10-20 years. These individuals would be in need of liver transplantation [1]. Meanwhile effective antiviral therapy has become a good option to prevent end stage liver disease and reduce morbidity and mortality. Pegylated interferon (PEG-IFN) and ribavirin (RBV) is the standard of care for chronic hepatitis C. Genotype has shown to be the most important factor in treatment outcome [2]. Sustained virological response rates (SVR) of up to 50% and >75% can be achieved in patients infected with HCV genotype 1/4, with genotype 2/3, respectively. For patients infected with genotype (GT) 1 a treatment course of 48 weeks with combination therapy (PEG-IFN and RBV 1000-1200 mg/day) is recommended [3]. Patients infected with genotype 2 or 3 can be treated with a lower dose of RBV (800 mg/day) for a shortened duration of 24 weeks without loss of efficacy [4]. Based on these data treatment guidelines for chronic hepatitis C recommend the use of a fixed dose of 800 mg/day, since it is better tolerated and as effective as the 1,000 or 1,200 mg per day [2]. Treating patients is still a challenge due to the high rate of adverse events [3,5]. Among the adverse events of treatment RBV induced haemolytic anaemia is of particular importance as it accounts for up to 36% of HCV treatment interruptions, therefore treatment regimes with lover RBV doses and hence better tolerated could help to improve tolerability and acceptance of antiviral treatment [6]. In the era of new antiviral HCV treatment with direct acting antivirals and additional side effects like e.g. anaemia it might be important to define the importance of RBV better.

There are several differences between patients infected with various genotypes. For example patients with genotype 3 typically have a virus induced hepatic steatosis. Both genotype 2 and 3 patients respond much better and faster to interferon therapy and have higher SVR rates (genotype 2 around 90%, genotype 3 around 70%). The pivotal trial of Hadziyannis [4] clearly showed that genotype 2/3 patients need less RBV than GT 1 patients. The reason for this difference is unknown. Several attempts were made to make treatment more tolerable in patients with GT 2/3 infection by conducting trials either to reduce the length of treatment [7], the dose of PEG-IFN [8] or of RBV. Treatment duration could be shortened to 12-16 weeks if a higher dose of RBV (1,000/1,200 mg/day) was used without considerable loss of responsiveness or increased risk of relapse [7,9,10]. On the other hand we have shown in a randomized, controlled trial conducted 2003-2006 that in patients with chronic hepatitis C, genotype 2/3 the dose of RBV can be reduced to 400 mg/day if treated for 24 weeks [11] without an increase in relapse rates. Consecutively the next logical step was to try also to lower the treatment duration to just 16 not 12 weeks, which would favour a RBV dosage of 1000 mg/day. With the slightly longer treatment course we hypothesised a reduced RBV dosage of 400 mg/day would lead to comparable treatment results and matchable relapse rates. The hope was to further define the role of RBV in patients infected with genotypes 2/3.

## Methods

Criteria for patients being included in the trial were age 18 to 65 years, chronic hepatitis C, HCV genotype 2 or 3 infection, quantifiable HCV RNA in serum (>600 IU/mL by Cobas Amplicor HCV Monitor Test v.2.0) and elevated serum ALT activity (>1.5 times the upper limit of normal [ULN] in the previous 6 months and during screening). Further inclusion criteria for patients were a haemoglobin value ≥ 12 g/dL (women) or ≥ 13 g/dL (men), a leukocyte count >3,000/μL, a platelet count ≥ 100,000/μL, and a serum creatinine level <1.5 times the ULN. Pre-menopausal women had to have a negative pregnancy test within 24 hours of the first dose. All fertile patients were advised to use two forms of effective contraception during and until 6 months after the end of treatment. Patients who had received prior treatment with IFN or RBV were excluded. Other exclusion criteria were co-infection with hepatitis B, immunodeficiency virus, decompensated liver disease of any cause, coronary heart disease, diabetes mellitus requiring insulin therapy, autoimmune disorders, active alcohol or drug addiction and/or any other unstable chronic medical condition. Patients on an opiate substitution therapy were suitable for the trial.

This clinical trial has been reviewed and approved by the ethics committees from the medical university Vienna and Elisabethinen Hospital Linz.

### Study Protocol

The current study is based on an amendment to a previously published study comparing two treatment groups: Group A received 180 μg Peginterferon2a/week in combination with 800 mg/d of RBV and group B received 180 μg Peginterferon2a/week in combination with 400 mg/d of RBV for a duration of 24 weeks. The protocol amendment expanded the initial comparison of the groups A and B by two further treatment groups: 180 μg Peginterferon2a/week in combination with either 800 (group C) or 400 mg/d (group D) for 16 weeks. As depicted in the amendment the randomization for group A as a control group was continued whereas the randomisation for group B was terminated. Suitable patients for this study protocol amendment were randomized 2:2:1 (C: D: A) after entering a 35-day screening phase. Randomization was performed centrally and was stratified according to HCV genotype (2 versus 3), baseline HCV RNA level (≤800,000 IU/mL versus >800,000 IU/mL) and study centre. The ethics committees of all participating study centres approved the protocol and patients signed a written informed consent before screening. For safety reasons an interim analysis was required 12 months after the first patient was randomized within the amendment. The unfavourable results of this interim analysis prompted an early termination of the study for ethical reasons. In case of laboratory abnormalities PEG-IFN dosage could be reduced from 180 to 135, 90 and 45 μg/week preferably in a stepwise manner autonomously by the investigator. The dosage reduction or discontinuation procedure for RBV concerning haemoglobin values was as follows: values of >8.5 and <10 g/dL induced a reduction of 200 mg/day; values of <8.5 g/dL or values <10 g/dL despite four weeks of treatment at a reduced dosage of 600 mg/day resulted in discontinuation of RBV. In patients with stable coronary heart disease any decrease >2 g/dL over a four week period resulted in a dosage reduction of 200 mg/day. The dose of RBV could be reduced in 200 mg/day steps in patients randomized to group D. Any dose reduction could be reversed to restore the originally assigned dose if the event causing the reduction terminated. Monotherapy with PEG-IFN could be administered if RBV was discontinued. However monotherapy with RBV was not permitted. Erythropoietin-stimulation was allowed at the investigator's decision if haemoglobin levels fell below 10.5 g/dL.

The primary efficacy end point was sustained virological response (SVR), defined as undetectable HCV RNA (<50 IU/ml) by qualitative PCR assay (Cobas Amplicor HCV Test v.2.0) after 24 weeks of untreated follow-up (week 40 or week 48).

Patients in the groups with the shortened treatment regimen experiencing a relapse in the follow up phase were offered an off-study retreatment with standard therapy of 24 weeks PEG-IFN+RBV 800 mg/day. Nonresponders at week 16 were retreated by choice of the investigator.

### Statistical and Analytical Methods

#### Sample Size Calculation

The original trial comparing 800 mg/d RBV (group A) with 400 mg/g (group B) [11] for 24 weeks of treatment assumed response rates of 78% in both treatment groups with a maximum acceptable difference of 15%. Similar assumptions for the comparison of group C vs. D resulted in a planned sample size of 144 patients per group. The reference group (group A) was continued with an allocation ratio of 1(group A):2(group C):2(group D) using a dynamic randomization protocol and ultimately resulting in a planned sample size of 360 patients in total (A + C + D) for the amendment phase. A sample size of 244 genotype 3 patients was considered to be sufficient to compare the sustained response rates with a power of 80% and a significance level of 5%.

Due to the early termination of the study, the incomplete randomization process as well as the stratification by study centre, genotype and viral load the patients were unevenly distributed between the study groups (A = 7; C = 31; D = 51). The use of a dynamic randomization procedure and the fact that because in the original trial 141 patients have already been randomised to group A the allocation of patients to the groups C and D in the beginning of the amendment phase was favoured [12]. Dynamic randomization methods allocate patients to treatment groups by checking if similar patients were already randomized, and allocating the next patient to balance the treatment groups best across all stratification variables.

### Statistical Analysis

For safety reasons an interim analysis was required 12 months after the first patient was randomized within the amendment phase. Originally the non-inferiority of PEG-IFN+RBV 800 mg/d versus PEG-IFN+RBV 400 mg/d for 16 weeks should have been investigated but due to the premature termination of the study for ethical reasons (see results) the groups were analysed according to statistical difference using the Fisher's exact test. Additional comparison such as baseline characteristics was done using Chi^2^-Test for nominal and using Mann-Whitney-U-Test for quantitative variables. Due to low numbers the control group (group A) was not analyzed.

## Results

**Figure 1:**
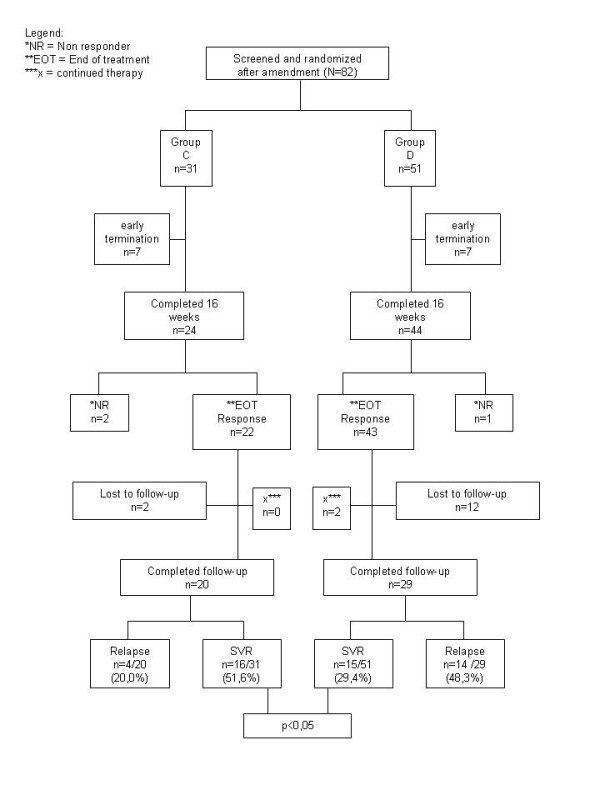
**Patient flow**.

The study groups were comparable in the baseline characteristics age, sex, BMI and route of transmission (Table 1). The viral load was higher in the group C (1.4 Mio IU/ML) compared to group D (0.87 Mio IU/mL) but not statistically significant (p = 0.43). 14 patients terminated treatment early (group C: 7; group D: 7) because of withdrawal of consent (C = 4, D = 2), drop out (C = 2, D = 3), change of therapy regimen (C = 1, D = 1), inaccurate randomization (D = 1). Premature treatment discontinuation was higher in patients who acquired HCV infection via intravenous drug usage (IVDU) (46.9% vs. 21.2% in non IVDU). At an interim analysis 12 months after the first patient was randomized an inferior outcome of group D (400 mg RBV/d) as compared to group C (800 mg/d) was noted. Therefore the study was prematurely terminated for ethical reasons. At this time 89 patients were already enrolled (group C: 31, D: 51, control: 7). A flowchart showing all patients of groups C and D is given in Figure 1.

**Table 1:** Baseline characteristics of all randomized patients

	Group C (ribavirin 800 mg)	Group D (ribavirin 400 mg)	All patients
n	31	51	82

Age, years ± SD	36.6 ± 11.0	34.9 ± 9.9	35.5 ± 10.3

Sex, male/female	13/18	21/30	34/48

Mean baseline HCV RNA (Mio. IU/mL) ± SD	1.447 ± 2.93	0.869 ± 1.46	1.083 ± 2.13

Mean Body weight (kg) ± SD	74.2 ± 18.8	68.7 ± 13.9	70.8 ± 16.0

Mean BMI ± SD	24.5 ± 5.7	23.3 ± 3.6	

**Means by which HCV was acquired, n (%)**			

Blood transfusion	3 (9.7)	6 (11.8)	9 (11)

Intravenous drug use	20 (64.5)	28 (54.9)	48 (58.5)

Other	3 (9.7)	2 (3.9)	5 (6.1)

Unknown	5 (16.1)	15 (29.4)	20 (24.4)

### Efficacy

According to an intention-to-treat analysis the overall SVR rate in the whole study group was 37.8%. The SVR rate was statistically different in the study groups with 51.6% in group C and 28.4% in group D (p = 0.038). The end of treatment response rate (EOT) was 64.5% in group C and 56.9% in group D. The relapse rate of patients who completed follow up was 20% in group C and 48.3% in group D. Patients with low viral load (≤800,000 IU/mL) had higher SVR rates (C: 67%, D: 33%) than those with high viral load (>800,000 IE/mL) (C: 33%, D: 21%).

The SVR rate of those who acquired HCV infection via IVDU was 32.7% compared to 45.5% in patients without IVDU history. In group C the SVR rate in patients without IVDU history was 72.7% compared to 31.8% in group D. The baseline characteristics according to virological response are presented in Table 2.

**Table 2:** Baseline characteristics of patients according to virological response

	SVR	relapse	NR
Mean baseline HCV RNA (Mio IU/mL)	0.76 ± 1.58	1.84 ± 3.28	0.96 ± 0.58

Mean Body weight (kg) ± SD	69 ± 13	76 ± 18	71 ± 8

Mean BMI ± SD	23 ± 4	25 ± 5	24 ± 2

Mean Age, years ± SD	34 ± 9	44 ± 9	50 ± 16

Sex, male/female ± SD	17/14	14/4	1/2

Looking only at genotype 3 patients (n = 70), which were the majority of patients; the SVR rates were similar to the overall data with a SVR rate of 53.8% in group C and 29.6% in group D. The relapse rates of patients with completed follow-up were 17.7% in group C and 48.0% in group D.

### Safety

The two different regimens were well tolerated with similar adverse event rates in both groups. The most common adverse events were flu-like syndrome, fatigue and/or epigastric discomfort which were reported by 70.9% in group C and by 64.7% in group D. Psychiatric events such as depression were seen by 38.7% in group C and by 45.1% in group D. Pruritus and/or exanthema were seen in 25.8% in group C and in 31.4% in group D. In group C 12.9% experienced hair loss and in group D 13.7%. Thyroid hyper- or hypo function was observed in 6 patients (group C: 1; group D: 5).

**Figure 2:**
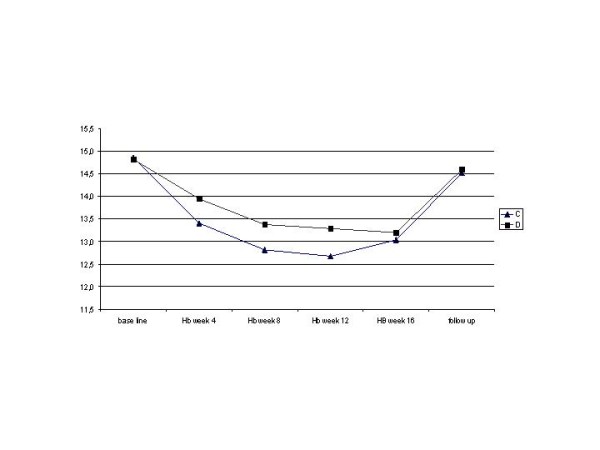
**Mean haemoglobin concentration during treatment and follow up**.

The frequency of laboratory abnormalities was comparable in both treatment groups. Only the haemoglobin level was lower in patients receiving RBV 800 mg/day than those receiving 400 mg/day (see Figure 2). PEG-IFN alfa-2a was reduced in 16.1% (5 patients) in group C and in 9.8% (5 patients) in group D. Three of five patients with PEG-IFN alfa-2a dose reductions in group C achieved SVR whereas two relapsed and in group D two of five patients achieved SVR, one patient did not respond to the therapy, one patient also experiencing a RBV dosage reduction relapsed and one patient was lost to follow up. In total four patients received any haematopoietic growth factor. One patient received erythropoietin, two patients received granulocyte colony-stimulating growth factor and one patient received both which was permitted in the study protocol. There were two reductions in the dosage of RBV which were surprisingly both conducted in the 400 mg/day treatment group. The two reductions of RBV resulted in one relapse and one patient was lost to follow up.

## Discussion

While reducing RBV dose in patients treated for 24 weeks did not affect the efficacy of treatment [11], the results of this follow up study indicate that shortened treatment duration with a reduced RBV dosage is associated with lower SVR rates mostly due to high relapse rates. The results support also the findings of Shiffman [13] that shortened treatment duration leads to inferior results. In that study shortening of treatment to 16 weeks was only feasible in patients with low baseline viral load. While the mean baseline viral load in the present study was lower in Group D (400 mg/d RBV) than in group C (800 mg/d RBV), SVR rates were lower in patients in group D than in group C. Thus, shortening treatment duration in patients receiving RBV dosages below the recommended dosage is not feasible.

In contrast, Mangia reported that in patients receiving weight based RBV treatment duration can be shortened to 12 weeks [7]. Further analysis of predicting factors allowing treatment shortening revealed that the requirements to shorten therapy were a rapid virological response (RVR), absence of advanced fibrosis, low baseline viremia, and a low BMI as well as genotype 2 [10,14,15]. Based on our data a RBV dosage of at least 800 mg/day is essential when shortening of treatment is considered. In case of favourable baseline factors treatment duration can be shortened but if there is need for RBV dose reduction it is essential for the patient to undergo the full duration of 24 weeks of antiviral treatment. However, if genotype 2/3 patients achieve RVR a 16 week course of treatment with a weight based RBV dosage seams equivalent to 24 weeks of treatment with 800 mg/day of RBV [16].

The mechanisms of RBV in treatment of hepatitis C are still a matter of ongoing discussion. A recent study suggests that RBV augments the induction of interferon-stimulated genes in patients treated for HCV infection[17]. The main effect of RBV is to prevent relapses in end of treatment responders. However RBV is not the only factor required for sustained viral elimination, duration of treatment is also very important. RBV appears to have little if any effect in the early virologic response in GT 3 patients [18]. The results of this study reflect the interplay between of RBV dose and length of treatment. The recent discovery of IL28B polymorphism [19-21] has lead to a better understanding of the course of viral elimination on PEG/RBV therapy. Relapse rates are substantially lower in C/C patients than in T-allele carriers. Prolongation of treatment in GT 2/3 patients was able to decrease relapse rates in T allele carriers [22]. Thus, in future treatment individualization based on the IL28B genotype may help to select the best duration of treatment, as well as the required dose of PEG-IFN and RBV.

Although the mean haemoglobin level was lower in patients randomized to 800 mg RBV than to 400 mg RBV per day the dose reductions were noted only in group D suggesting that the tolerability cannot generally be improved by a reduced RBV dosage. The study results show that adverse event rates do not justify a reduced RBV dosage since they were comparable in both groups. However, in a recent study it has been shown that two functional variants in the inosine triphophastase (ITPA) [23] gene are protective against treatment related anaemia. These genetic biomarkers make patients more or less sensitive to RBV induced anaemia. The clinical utility of ITPA genotyping remains unclear, it may be important in patients with high risk of RBV associated anaemia or related mortality, but until now there is no evidence that ITPA variants are associated with the need of RBV dose modifications or treatment outcome in genotype 2/3 patients [23,24]. Thus, RBV in the standard dosage is still a backbone of antiviral therapy.

An important factor contributing to the unfavourable results was a high proportion of premature treatment discontinuation especially in patients who acquired HCV infection via IVDU (46.9% vs. 21.2%). Adherence rates range from 37 [25] to 76% and up to one third of patients is lost to follow up [26] and seem to be somewhat better than in our cohort. Therefore patients' selection seems to be crucial in treating patients with history IVDU another point might be problems at the less experienced sites managing these patients [27]. As shown in recent studies anti HCV treatment is effective in IVDU patients. Baseline factors associated with lower SVR included decreased social functioning and current opiate pharmacotherapy. Adherent participants were more likely to achieve SVR (63% vs 29%; P = .025) [28,29]. As shown by Ebner it is important to select stable patients who are able to complete therapy but being too strict in the selection process excludes up to 80% of patients with a fair chance of being cured but overall SVR rates might be reduced [30] as high adherence can be achieved in patients with a history of IVDU [31].

### Limitations of the study

The number of patients is small; the study was terminated early because of unfavourable treatment results in the low RBV group at an interim analysis. Due to the early termination of the study, the incomplete randomisation process as well as the stratification by study centre, genotype and viral load the patients were unevenly distributed between the study groups and this reflects clearly a limitation of the study. The high proportion of premature treatment discontinuation in patients who acquired HCV via IVDU might reflect poor patients' selection or problems in managing these patients in less experienced centres.

## Conclusion

Both treatment duration and the dose of RBV play a major role to optimize outcome of patients with GT3. If one intends to shorten the treatment weight based RBV dose should be used, if lower RBV doses are used patients should be treated for at least 24 weeks as. A treatment regimen with a reduced RBV dosage and shortened treatment duration is associated with low SVR rates due to high relapse rates.

## Competing interests

Peter Ferenci is a member of the global advisory board and of the speaker's bureau of ROCHE, Basel CH and Rottapharm-Madaus, Monza, Italy. He is also advisor to Böhringer-Ingelheim, Vertex/Tibotec, Pfizer and MSD Austria. He receives also an unrestricted research grant from ROCHE Austria. Andreas Maieron, Michael Strasser, Michael Geschantler and Thomas Scherzer serve as speakers for Roche Austria and MSD Austria. All other authors have no financial disclosures to report.

## Authors' contributions

PF designed the study. SM, AM, TS, HL, GF, MB, MG treated the patients, collected and assembled data, SM and AM carried out the data analysis and the data interpretation. AM and SM wrote the manuscript. The manuscript was finally approved by all authors
